# Differences Between the Prenatal Effects of Fluoxetine or Buspirone Alone or in Combination on Pain and Affective Behaviors in Prenatally Stressed Male and Female Rats

**DOI:** 10.3389/fnbeh.2019.00125

**Published:** 2019-06-11

**Authors:** Irina P. Butkevich, Viktor A. Mikhailenko, Elena A. Vershinina, Gordon A. Barr

**Affiliations:** ^1^Laboratory of Ontogenesis of the Nervous System, I.P. Pavlov Institute of Physiology, Russian Academy of Sciences, St. Petersburg, Russia; ^2^Department of Normal Physiology, State Pediatric Medical University, St. Petersburg, Russia; ^3^Department of Information Technologies and Mathematical Modeling, I.P. Pavlov Institute of Physiology, Russian Academy of Sciences, St. Petersburg, Russia; ^4^Department of Anesthesiology and Critical Care Medicine, The Children’s Hospital of Philadelphia and the Perelman School of Medicine at the University of Pennsylvania, Philadelphia, PA, United States

**Keywords:** fluoxetine, buspirone, combination of the drugs, prenatal stress, pain, depression, adolescent rat, sex differences

## Abstract

The selective serotonin reuptake inhibitor fluoxetine and the 5-HT1A receptor agonist buspirone are used to treat depression and anxiety. Previously we demonstrated that chronic stress during pregnancy (prenatal stress) in rats, used as a model of maternal depression risk, increased inflammatory pain and depressive-like behavior in the offspring; buspirone injected to pregnant dams was protective. Clinically, the addition of buspirone to fluoxetine increases the latter’s efficacy in treating depression in patients. Here, we investigated the influence of repeated prenatal injections of fluoxetine, buspirone or their combination on pain- and depressive-like behaviors in prenatally stressed young male and female rats. Prenatal stress augmented depressive-like behavior and both thermal and inflammatory pain (formalin test), replicating our prior findings, and increased basal levels of corticosterone in the blood plasma. Both drugs and their combination reduced the effects of prenatal stress on thermal pain and depressive-like behavior independently of sex. The combination of fluoxetine and buspirone, compared with fluoxetine, was more antinociceptive in the hot plate test in both sexes, and when compared with buspirone, was more antinociceptive only in males. A detailed study of the time-course of formalin-induced pain showed a nuanced effect of these drugs that was sex-dependent. The combination of the two drugs was less effective in females than males during the initial acute phase of nociceptive behavior in flexing + shaking behaviors, whereas that combination was more effective than fluoxetine alone in the first acute phase of licking behavior in females. The antinociceptive effect of buspirone dominated that of the drug combination and of fluoxetine alone, especially during the interphase of the formalin test in both sexes for both flexing + shaking and licking, suggesting a more effective prenatal action of buspirone on the development of a descending serotonergic inhibitory system modulating pain in the spinal cord dorsal horn neurons. Our results indicate that inflammatory pain-like responses integrated at the spinal level in males were more vulnerable to prenatal stress than females. In licking, the antinociceptive effect of fluoxetine and drug combination in the interphase was more in males than females. The data underscore the importance of considering sexual dimorphism when using drug therapy.

## Introduction

Clinical and experimental studies have found that strong and/or persistent stress during pregnancy (prenatal stress) is associated with lasting dysfunction in the central nervous system (CNS) that increases vulnerability towards affective disorders (Weinstock, 2010; Entringer et al., 2015; Kundakovic and Jaric, 2017; Scheinost et al., 2017; Hartman and Belsky, 2018; Huizink and de Rooij, 2018). In addition to increased risk of affective disorders, prenatal stress alters other neurobehavioral functions, including pain perception. Our knowledge of prenatal stress effects on reactivity of the pain, however, is limited (Sternberg and Ridgway, 2003; Sun et al., 2013; Knaepen et al., 2014). In animal studies, maternal stress replicates risk factors for depression as well as other psychosocial disorders (Weinstock, 2008, 2017). Prenatal stress can influence the offspring’s neurodevelopment *via* multiple ways. Prenatal stress alters serotonergic function (Van den Hove et al., 2006; Gemmel et al., 2018; Kiryanova et al., 2018; Soares-Cunha et al., 2018), the hypothalamo-pituitary-adrenal axis (Gemmel et al., 2017; Morsi et al., 2018), GABA-ergic (Nejatbakhsh et al., 2018), and glutamatergic systems (Cattane et al., 2018; Lin et al., 2018) and immune system function (Bittle and Stevens, 2018; Goldstein et al., 2019).

The serotonin 1A receptor subtype (5-HT1AR) and glucocorticoid receptors (GR) are thought to be the main targets of prenatal stress (Van den Hove et al., 2006; Kiryanova et al., 2018). The serotonergic system and the HPA axis are closely interrelated (Andrews and Matthews, 2004; Wyrwoll and Holmes, 2012). GR are on neurons in the CNS regions classically associated with nociception and there is evidence that HPA axis directly influences nociception, particularly pre- and perinatally (Shagura et al., 2016; Zouikr et al., 2016). In the last week of the rat fetal development, the levels of corticosteroids in the blood increase, peaking 1 day before term (Waddell and Atkinson, 1994). The expression of 5-HT1AR first appears in the rat during the initial stages of embryonic development of the hippocampus (Patel and Zhou, 2005). 5-HT1AR is highly expressed in the limbic system, prefrontal cortex (PFC), raphe nuclei, and spinal cord (Popova and Naumenko, 2013). The former two CNS structures are of particular relevance in affective behavior and the etiology of depressive disorders (Liu et al., 2017), and the latter two, in pain processing and its modulation (Wang and Nakai, 1994). Neuroanatomical and functional connections among these structures determine the integration of nociceptive and affective signals and the involvement of the descending serotonergic system that regulates nociceptive signals in pain and depressive behaviors (Chaouloff, 2000). Since 5-HT1AR is involved in nociception (Granados-Soto et al., 2010) and psycho-emotional behavior (Savitz et al., 2009), changes in its activity in the prenatal period may manifest itself later in alteration of various types of adaptive behaviors (Knaepen et al., 2013, 2014; Kiryanova et al., 2017, 2018; Pawluski and Gemmel, 2018).

There is increasing evidence to suggest that the 5-HT1AR is involved in depression and the actions of antidepressant drugs (Savitz et al., 2009; Carr and Lucki, 2011; Richardson-Jones et al., 2011) and is an important target for the pharmacological treatment of disorders in the CNS (Lacivita et al., 2008; Berrocoso and Mico, 2009; Albert and Fiori, 2014; Turcotte-Cardin et al., 2019). Of the selective serotonin reuptake inhibitors (SSRIs) used for the treatment of depression, fluoxetine is among those recommended for pregnant women (Kaihola et al., 2016). SSRIs cross the placental barrier (Pohland et al., 1989; Ewing et al., 2015), bind to the serotonin transporter (SERT) and block the presynaptic reuptake of serotonin (5-HT), thus increasing the level of 5-HT in the synaptic gap (Kiryanova et al., 2013). Since 5-HT is a key regulator of early developmental processes in the CNS (Lauder, 1990), disruption of 5-HT balance in the fetus can affect its development and lead to altered adaptive behavior in later life. However, fluoxetine does not reduce the level of depression in all patients, so attempts have been made to improve its efficacy. A number of clinical observations in adult patients suffering from depression suggest that the combination of 5-HTIA receptor agonists with SSRI’s improves therapeutic outcomes (Pierz and Thase, 2014; Wang et al., 2015). For instance, the antidepressant vilazodone (Stuivenga et al., 2019) integrates properties of a partial 5-HT1A receptor agonist with SERT blockade. Despite a number of positive results with the use of SSRIs in combination with agonists of 5-HT1A receptors in adult patients, there continues to be a need for a systematic study of results of the influence of such types of combination in animal models (Stuivenga et al., 2019). It should be noted that our knowledge of the possible antinociceptive effect of SSRIs (Dharmshaktu et al., 2012; Zammataro et al., 2017; Barakat et al., 2018; Hamdy et al., 2018), as well as buspirone (Giordano and Rogers, 1992; Pavlaković et al., 2009; Haleem et al., 2018) is limited, and the available data are inconsistent.

Buspirone is widely used for treating generalized anxiety disorder (Albert and François, 2010; Albert and Fiori, 2014; Howland, 2015; Wilson and Tripp, 2018). It is a full agonist at presynaptic 5-HT1A autoreceptors, where it initially inhibits synthesis and release of 5-HT. Repeated administration of buspirone inhibits the function of 5-HT1A autoreceptors and its feedback control over the synthesis and release of 5-HT (Haleem et al., 2018). Buspirone is also a partial agonist at 5-HT1AR in the hippocampus and frontal cortex, where it is expressed as a heteroreceptor on GABAergic and glutamatergic neurons, and helps attenuate dysfunctional serotonergic transmission in depressed patients (Celada et al., 2013). Our previous work found that buspirone injected to pregnant rat dams that were stressed during pregnancy, attenuated the inflammatory pain response in the formalin test in the offspring of those dams (Butkevich and Vershinina, 2001).

The formalin test is widely used for assessment of antinociceptive effect of the drugs and induces reproducible and quantifiable pain behavior in two distinct phases with an interphase between them (Dubuisson and Dennis, 1977; Barr, 1998). The first phase represents acute pain and is mediated in part by AMPA receptors whereas the second phase is thought to be more inflammatory and is mediated in part by NMDA receptors. The interphase is a period of dampened pain and is a transition between these two phases and may be mediated by descending inhibition from medullary sites to the spinal cord dorsal horn (Shields et al., 2010; Fischer et al., 2014, 2015; Ishikura et al., 2015; Urien et al., 2017). The formalin test is often used to study the different mechanisms of analgesia induced by various new drugs and the acute to chronic pain transition (Price et al., 2018; Zhang et al., 2018).

Most studies of prenatal exposure on behavior have so far focused on male offspring. Nonetheless, it is essential to include individuals of both sexes in research studies, especially given the sex differences in psychological disorders (Aloisi, 2017; Kundakovic and Jaric, 2017; Gemmel et al., 2019). Data on sex differences in response to SSRIs for the management of chronic pain are limited. We included rats of both sexes in our investigations to determine better whether mechanisms responsible for the effects of fluoxetine and buspirone are sex-specific, which is important for subsequent drug development. Previously, we showed that the chronic administration of fluoxetine to pregnant rat dams that were not stressed during pregnancy did not alter formalin-induced pain behavior or the level of depressive-like behavior in the forced swim test in adolescence (Butkevich and Mikhailenko, 2018). In the present study, we injected the drugs to pregnant rat dams stressed during pregnancy. In view of the questions raised in the literature cited above, the present study compared the prenatal effects of a combination of fluoxetine and buspirone and of each drug alone on basal thermal pain, inflammatory pain-like behavior, and affective behavior. Based on the available clinical literature, we hypothesized that the prenatal effect of the combination of fluoxetine and buspirone would be more effective than of fluoxetine or buspirone alone in altering pain- and depressive-like behaviors of the offspring of dams that were exposed to prenatal stress. We tested this hypothesis in peri-adolescent male and female rats, during an important developmental epoch in which depression is often first evidenced and an age that we have previously studied (Butkevich and Vershinina, 2001; Butkevich et al., 2017).

## Materials and Methods

### Animals

All experimental procedures were approved by the Local Ethics Committee for Animal Experiments of the I. P. Pavlov Institute of Physiology, Russian Academy of Sciences (St. Petersburg, Russia) and followed the guidelines published by the Committee for Research and Ethical Issues of the IASP on ethical standards for investigations of experimental pain in animals. Sixty-seven adult female and 33 male Wistar rats (180–220 and 270–300 g, respectively) were obtained from the vivarium of the I.P. Pavlov Institute of Physiology. The work was performed on animals from the biocollection of Pavlov Institute of Physiology of the Russian Academy of Sciences. After 2 days of adapting to new quarters, the rats were mated, and a vaginal smear was examined next morning to verify insemination. The days of insemination and birth were considered as gestational day (GD) 0 and postnatal day (PD) 0, respectively. Pregnant dams were housed four per cage, then individually after the 17th day of pregnancy. All animals were maintained under standard conditions (12 h light, 12 h dark, lights on at 08:00, 20–22°C) in standard plastic rat cages with food and water available *ad libitum*.

### Experimental Design

On GD nine rat dams were randomly assigned to the following groups with daily morning intraperitoneal injections until delivery: (1) vehicle + vehicle (labeled “V”), 0.5 ml/kg, *n* = 21); (2) vehicle + fluoxetine (labeled “F”), Sigma, 10 mg/kg, *n* = 14); (3) vehicle + buspirone (labeled “B”), Sigma, 3.5 mg/kg, *n* = 11); or (4) combination of fluoxetine and buspirone (labeled “FB”) at the doses listed above, *n* = 21). The doses of buspirone and fluoxetine were chosen according to the literature and our previous studies (Kim and Druse, 1996; Mikhailenko and Butkevich, 2019). It should be noted that at the dose used here, the existing literature indicates that buspirone does not alter dopamine or norepinephrine function (Cimino et al., 1983). Nine dams of the control group (V) were unstressed during pregnancy (“PNS”). All other rats were exposed to restraint stress from GD 15 to delivery. Stress during pregnancy in rodents (prenatal stress, “PS”) is thought to be a model of maternal depression risk (Weinstock, 2008, 2017). For restraint stress, each dam was placed in a prone position in a cylinder, which was adjusted individually to the size of each pregnant dam, such that her movements were sharply restricted. This was done an hour twice daily, once in the morning (between 09.00 and 13.00) and once in the evening (between 14.00 and 19.00) in bright light. Dams were checked for the birth of litters twice daily at 9 AM and 7 PM. On PD 1, litters were culled to four males and four females. There were the following experimental groups of male and female rat offspring respectively: V-PNS *n* = 9, *n* = 9, V-PS *n* = 12, *n* = 11, F-PS *n* = 14, *n* = 12, B-PS *n* = 11, *n* = 9, FB-PS *n* = 21, *n* = 14. One male and one female were taken from each litter (V-PNS, V-PS, F-PS, B-PS, FB-PS) for behavioral experiments and corticosterone analysis. The remaining animals were used for other studies.

### Behavioral Tests

When the rats were 25 days of age, basal pain sensitivity in one male and one female from each litter which was selected for experiments in this study was measured in the hot plate test. After that these animals were marked for identification. Weaning was carried out after testing in the hot plate in the rats of all the groups. Males and females from each litter were placed together in the cage without the dam until the end of the experiments. Next day, the inflammatory pain responses were evaluated in the formalin test. Three days later, depression-like behavior was evaluated in the forced swim test.

### Hot-Plate (HP) Test

A rat was placed on a metal surface maintained at 55°C and the latency for nociceptive responses was measured (shaking or licking the hind paw). To prevent tissue damage, we terminated the test if there was no response after 30 s. The response latency was averaged from three trials with 10 min intervals between each trial. The testing apparatus was thoroughly cleaned between trials.

### Formalin Test

The formalin test produces a very reproducible and quantifiable pain behavior represented by two phases. After plantar injection of formalin (2.5%, 1.0 μl) into the left hind paw, the rat was placed singly in a chamber (25 × 20 × 10 cm) with transparent glass walls for 60 min. The formalin response consists of flexing, shaking and licking behaviors organized in two phases with the period of the interphase, period of quiescence, between them. The first acute short phase (about 6 min) after the formalin injection is thought to arise from direct activation of myelinated and unmyelinated nociceptive afferent fibers. The second tonic phase (about 51 min) is considered to result from changes in CNS function induced by neural activity generated during the acute phase and from the developing inflammation caused by the formalin during the tonic phase. The formalin test allows studying mechanisms through which persistent nociception is generated (Dubuisson and Dennis, 1977; Abbott and Guy, 1995; Zhang et al., 2018). We recorded the number of flexes + shakes, organized at the spinal level, and duration of licking reaction, organized at the supraspinal level. We used a computer program specially created for our experiments to record, quantify and analyze the formalin-induced pain behaviors. Each 3-min value of flexing + shaking behavior and of licking duration was averaged and analyzed in the first phase (the first two 3-min periods; acute nociception), the interphase (the third 3-min period) and the second phase (the fourth to 20th 3-min periods; inflammatory pain response) of the formalin test, according to the literature (Taylor et al., 1998) and our previously published data (Butkevich et al., 2007). The time-course of formalin-induced pain was plotted for each group of rats.

### Forced Swim Test

Each rat was individually placed into the glass cylinder filled with water (diameter 25 cm, height 60 cm, 24°±1° C). The time of immobility (the rat only made movements necessary to keep the head above the water) was recorded during the 5-min test. This parameter, characterizing the level of depression, is widely considered a negative index of the animal’s ability to cope with stress (Burke et al., 2016). However, over the past few years, there is a trend to interpret the time of immobility rather as the expression of a coping strategy (Molendijk and de Kloert, 2019). We had considered this issue earlier (Mikhailenko et al., 2010). Now, we believe that the use of a varied test battery is necessary to fully characterize depression-like behavior in male and female rats.

### Corticosterone Determination

A basal level of corticosterone and the level of corticosterone in response to forced swimming were determined in separate groups of males aged 25 days (in each group *n* = 5–7). Basal blood samples were collected by rapid decapitation without anesthesia at 9 AM; stress blood samples were collected at 30 min after the forced swim test. The blood samples were centrifuged, and the plasma was kept at −20°C. The content of corticosterone in plasma was determined in duplicate by immune-enzyme analysis, using standard kits (“Xema-Medica Co” Cat No: K210R; Russia); the intra-assay coefficient was 3.8.

### Statistical Analyses

Mixed-model ANOVA’s were used to explore the influence of Time (3 min periods), Sex (males, females) and Treatment (vehicle and no stress, vehicle and stress, buspirone and stress, fluoxetine and stress, buspirone + fluoxetine and stress) for the Phase I (first two 3-min periods) and Phase II (the fourth to 20th 3-min periods) separately and a two factor ANOVA (Sex and Treatment) for the Interphase (the third 3-min period). Two-factor ANOVA’s were used to explore the influence of Sex and Treatment (vehicle and no stress, vehicle and stress, buspirone and stress, fluoxetine and stress, buspirone + fluoxetine and stress) on the hot plate latency and immobility time in the forced swim test. Prenatal Treatments conducted (vehicle and no stress, vehicle and stress, buspirone and stress, fluoxetine and stress, buspirone + fluoxetine and stress) are the same throughout here. *Post hoc* comparisons were made with Bonferroni multiple comparisons test. A one-factor ANOVA was conducted to explore the influence of Treatment on basal corticosterone level and corticosterone level after forced swim test, again followed by Bonferroni *post hoc* tests. Data analysis was carried out with the SPSS Inc. software. The results are expressed as mean ± standard error of the mean (SEM).

## Results

The chronic administration of fluoxetine during pregnancy did not change the duration of pregnancy in dams, but there was a slight decrease in the number of rat pups compared to the number of pups in the control group, as well as a single death both in the newborn rat pups and rat pups during the transition from dam feeding to independent feeding. It is worth noting a similar negative effect on these indicators was also caused by the stressful procedure itself of a chronic prenatal injection of saline. In newborn offspring of both sexes, prenatal administration of fluoxetine caused a slight decrease in body weight compared with the body weight of rats born to mothers with saline and body weight of intact animals, while the administration of buspirone did not cause significant changes in the body weight of newborn rats. At 25 days of age, the body weight of males with prenatal fluoxetine remained lower than that of males with prenatal saline and intact males, whereas there was no difference in body weight between females in the studied groups. All data on the effects of prenatal administration of fluoxetine and buspirone in the dam and her offspring have been published in our previous articles (Butkevich et al., 2017; Butkevich and Mikhailenko, 2018; Mikhailenko and Butkevich, 2018).

Details of the analyses are in Table 1 and summarized below. The results are summarized in schematic form in Table 2.

**Table 1:** Details of the statistical analyses.

**Hot Plate Test**
Treatment (*F*_(4,114)_ = 14.14, *p* < 0.001, *β*^2^ = 0.332
*Post hoc: Males and Females:*
V-PNS > V-PS,	*p* < 0.05
V-PS< B-PS,	*p* < 0.01
V-PS< FB-PS,	*p* < 0.001
F-PS< FB-PS,	*p* < 0.001
*Post Hoc: Females only*	
V-PS< B-PS,	*p* < 0.01
V-PS< FB-PS,	*p* < 0.001
F-PS< FB-PS,	*p* < 0.01
F-PS< B-PS,	*p* < 0.05
*Post Hoc: Males only*	
V-PNS > V-PS,	*p* < 0.05
V-PS< FB-PS,	*p* < 0.001
F-PS< FB-PS,	*p* < 0.05
B-PS< FB-PS,	*p* < 0.01
**Formalin test–Flexing and Shaking**
*First Phase*	
Time	*F*_(1,113)_ = 109.9, *p* < 0.001, *β*^2^ = 0.493
Time × Treatment × Sex	*F*_(4,113)_ = 3.87, *p* = 0.006, *β*^2^ = 0.120
Treatment	*F*_(4,113)_ = 3.2, *p* = 0.015, *β*^2^ = 0.102
Treatment × Sex	*F*_(4,113)_ = 2.94, *p* = 0.024, *β*^2^ = 0.094
*Interphase*
Treatment	*F*_(4,113)_ = 4.8, *p* = 0.001, *β*^2^ = 0.144
Treatment × Sex	*F*_(4,113)_ = 2.4, *p* = 0.053, *β*^2^ = 0.079
*Second Phase*
Time	*F*_(6,671)_ = 81.7, *p* < 0.001, *β*^2^ = 0.420
Time × Treatment	*F*_(24,671)_ = 1.9, *p* = 0.005, *β*^2^ = 0.064
**Formalin test–Licking**
*First Phase*
Time	*F*_(1,113)_ = 27.8, *p* < 0.001, *β*^2^ = 0.2
Treatment	*F*_(4,113)_ = 4.5, *p* < 0.01, *β*^2^ = 0.14
*Interphase*
Treatment	*F*_(4,113)_ = 3.7, *p* < 0.01, *β*^2^ = 0.12
Sex	*F*_(1,113)_ = 6.8, *p* < 0.01, *β*^2^ = 0.06
*Second Phase*
Time	*F*_(9,988)_ = 68.3, *p* < 0.001, *β*^2^ = 0.38
Treatment	*F*_(4,113)_ = 4.5, *p* < 0.001, *β*^2^ = 0.14
Time × Treatment	*F*_(36,988)_ = 2.0, *p* < 0.001, *β*^2^ = 0.068
**Forced Swim Test**
Treatment	*F*_(4,108)_ = 21.519, *p* < 0.001, *β*^2^ = 0.444
*Post Hoc: Males and Females:*
V-PNS< V-PS,	*p* < 0.001
V-PS > B-PS,	*p* < 0.001
V-PS > F-PS,	*p* < 0.001
V-PS > FB-PS,	*p* < 0.001
Exactly the same results for females and males separately.
**Corticosterone level**
Basal Treatment:	*F*_(4,22)_ = 6.8, *p* = 0.001, *β*^2^ = 0.55
*Post Hoc:*
V-PNS< V-PS	*p* < 0.05
V-PS > F-PS	*p* < 0.001
V-PS > B-PS	*p* < 0.05

**Table 2:** Influences of prenatal stress and the drugs on behavior in the forced swim test, hot plate test and formalin test.

Stress/No Stress	Groups	Forced swim test		Hot plate test		Formalin test											
						Flexing + Shaking						Licking duration					
						First phase		Interphase		Second phase		First phase		Interphase		Second phase	
No Stress	V-PNS	¬♂	¬♀	¬♂	¬♀	¬♂	¬♀	¬♂	¬♀	¬♂	¬♀	¬♂	¬♀	¬♂	¬♀	¬♂	¬♀
	♂ *n* = 9
	♀ *n* = 9
Stress	V-PS	♂***	♀***	♂*	♀*	♂**	¬♀	♂*	¬♀	♂***	¬♀	¬♂	♀*	¬♂	♀*	♂***	♀*
	♂*n* = 12
	♀ *n* = 11
	F-PS	♂***	♀***	¬♂	¬♀	♂+	¬♀	¬♂	¬♀	♂+	¬♀	¬♂	¬♀	¬♂	¬♀	♂+++	♀+
	♂*n* = 14
	♀ *n* = 12
	B-PS	♂***	♀***	♂**	♀**	♂^∧∧^	¬♀	♂^∧∧∧^	¬♀	♂^∧∧^	¬♀	♂^∧^	♀^∧∧^	¬♂	♀^∧^	♂^∧∧∧^	♀^∧∧∧^
	♂*n* = 11
	♀ *n* = 9
	FB-PS	♂***	♀***	♂***	♀***	♂###	¬♀	♂#	¬♀	♂##	¬♀	¬♂	♀##	¬♂	¬♀	♂###	♀###
	♂*n* = 21
	♀ *n* = 14

Note: the symbols define effects in females and males. The “¬” before the symbol means that there was no significant effect for that behavior for that sex. *, **, *** mean the effect of V-PS as compared to V-PNS; ^+^, ^+++^; ^∧^, ^∧∧^, ^∧∧∧^; ^#^, ^##^, ^###^ mean the effect of F-PS, B-PS, FB-PS, respectively, as compared to V-PS. V-PNS, vehicle, prenatally non-stressed; V-PS, vehicle, prenatal stress; F-PS, fluoxetine, prenatal stress; B-PS, buspirone, prenatal stress; FB-PS, fluoxetine, buspirone and prenatal stress. The data in the table have been analyzed separately for all indicators.

### Hot Plate (HP) Test (Figure 1)

#### Thermal Withdrawal Latency (Figure 1)

Stress alone enhanced thermal nociception significantly in males and females, and this was decreased significantly by buspirone and combination of the drugs in rats of both sexes. The effect of the both drugs combined was greater than that of fluoxetine in prenatally stressed males and females and greater than the effect of buspirone in prenatally stressed males.

**Figure 1:**
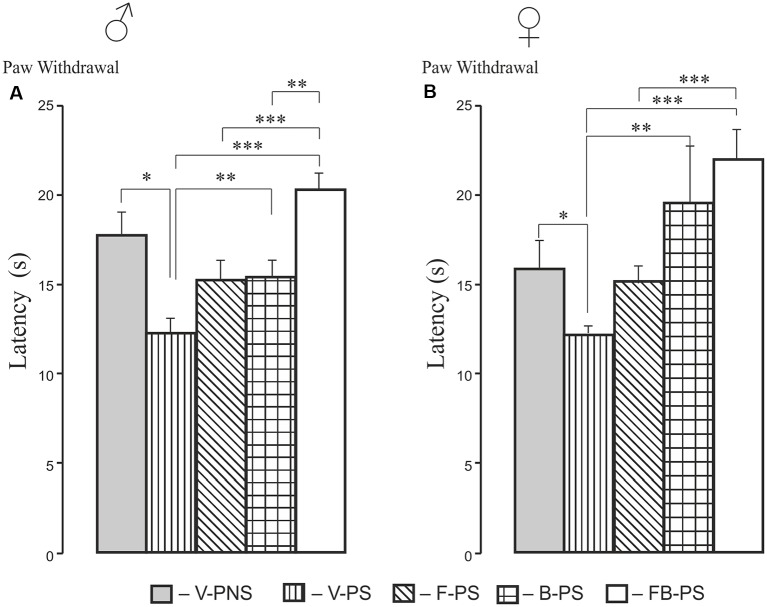
Effects of prenatal stress, fluoxetine, buspirone or fluoxetine and buspirone combination on paw withdrawal latency in the hot plate test in young male **(A)** and female** (B)** rats. V-PNS, vehicle without prenatal stress; V-PS, vehicle and prenatal stress; F-PS, fluoxetine and prenatal stress; B-PS, buspirone and prenatal stress; FB-PS, combination of fluoxetine and buspirone and prenatal stress. Vehicle (control), fluoxetine, buspirone or their combination were injected daily to pregnant dams from G9 to G20. Prenatal stress occurred from G15 to G20. *p < 0.05, **p < 0.01, ***p < 0.001. Data are means ± one standard error of the mean (SEM).

### Formalin Test (Figures 2, 3, 4)

#### Prenatal Stress Effects and Sex Differences (Figure 2)

**Figure 2:**
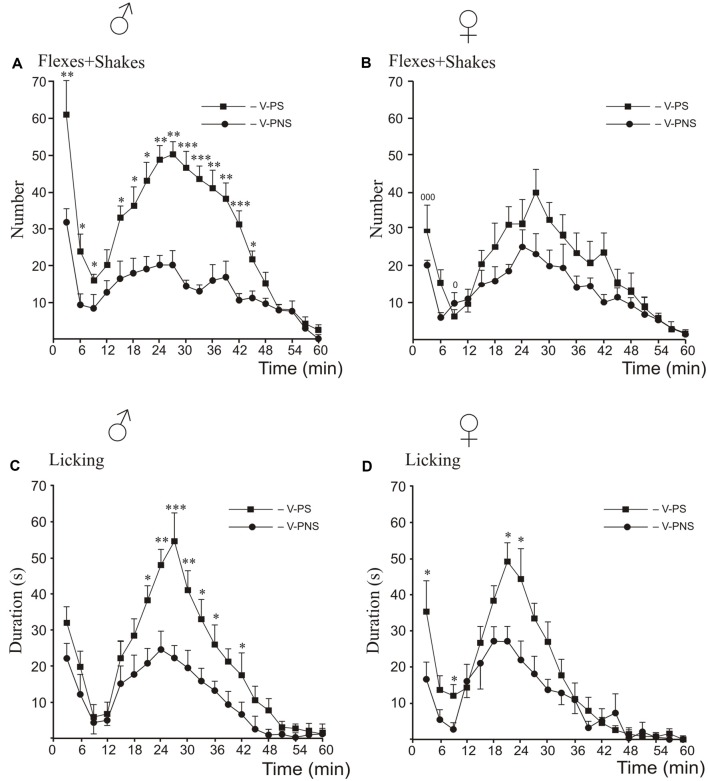
Effects of prenatal stress on the time-course of flexing + shaking behavior **(A,B)** and licking duration **(C,D)** in the formalin test during the first phase, the interphase and the second phase in young male **(A,C)** and female **(B,D)** rats. V-PNS, vehicle without prenatal stress; V-PS, vehicle and prenatal stress. For prenatal effects: Panels **(A,C,D)**: *p < 0.05, **p < 0.01, ***p < 0.001, V-PS vs. V-PNS. Panel **(B)**: no significant differences. *For sex differences in flexing and shaking in prenatally stressed young rats (panel* **B***)*: ^0^*p* < 0.05, ^000^*p* < 0.001. There were no sex differences for licking. Data are mean ± one SEM.

For flexing and shaking, there were sex differences in responding in the first phase and the interphase, with higher levels of responding in the males than the females (Figures 2A,B). The *post hoc* analysis of male responses revealed that prenatal stress increased flexing + shaking behavior in the first and the second phases, and the interphase (Figure 2A). There were no significant differences induced by prenatal stress in females in any phase (Figure 2B).

**Figure 3:**
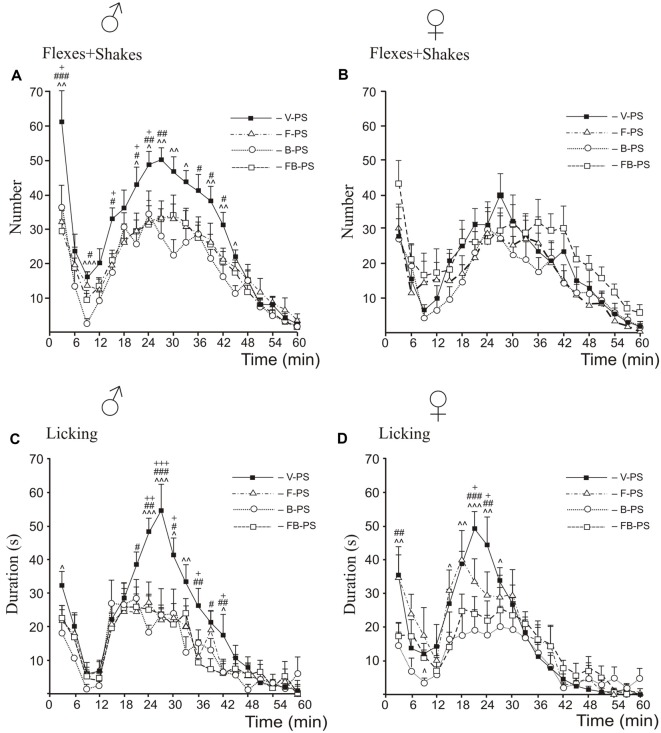
Effects of prenatal stress, fluoxetine, buspirone or fluoxetine and buspirone combination on the time-course of flexing + shaking behavior **(A,B)** and licking duration **(C,D)** in the formalin test during the first phase, the interphase and the second phase in young male **(A,C)** and female **(B,D)** rats. V-PS, vehicle and prenatal stress; F-PS, fluoxetine and prenatal stress; B-PS, buspirone and prenatal stress; FB-PS, fluoxetine and buspirone combination and prenatal stress. Panels **(A,C,D)**: F-PS vs. V-PS: ^+^*p* < 0.05, ^++^*p* < 0.01, ^+++^*p* < 0.001; B-PS vs. V-PS: ^∧^*p* < 0.05, ^∧∧^*p* < 0.01, ^∧∧∧^*p* < 0.001; FB-PS vs. V-PS: ^#^*p* < 0.05, ^##^*p* < 0.01, ^###^*p* < 0.001. Panel **(B)**: no significant differences. Data are mean ± one SEM.

**Figure 4:**
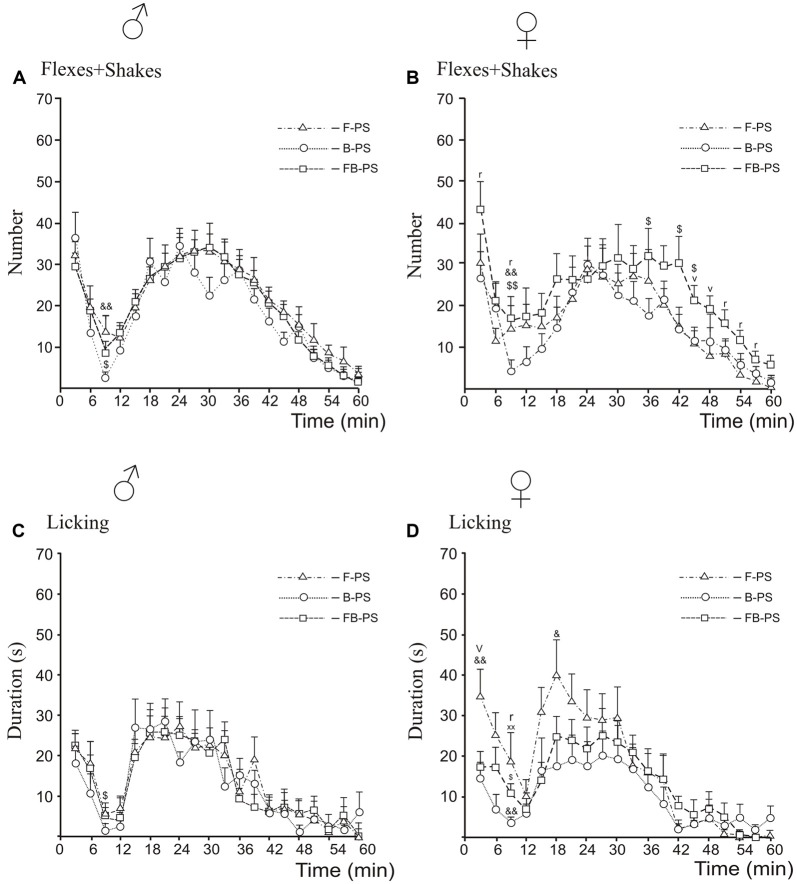
Differences between prenatal effects of fluoxetine, buspirone and fluoxetine and buspirone combination on the time-course of flexing + shaking behavior **(A,B)** and licking duration **(C,D)** in the formalin test during the first phase, the interphase and the second phase in young male **(A,C)** and female **(B,D)** rats. F-PS, fluoxetine and prenatal stress, B-PS, buspirone and prenatal stress, FB-PS, fluoxetine and buspirone combination and prenatal stress. For all panels: B-PS vs. FB-PS: ^$^*p* < 0.05, ^$$^*p* < 0.01; F-PS vs. B-PS: ^&^*p* < 0.05, ^&&^*p* < 0.01; F-PS vs. FB-PS: ^v^*p* < 0.05*. For sex differences in prenatal effects of fluoxetine and fluoxetine and buspirone combination in prenatally stressed young rats (panels* **B,D***)*: F-PS: ^xx^*p* < 0.01; FB-PS: ^r^*p* < 0.05. Data are mean ± one SEM.

For licking duration, the *post hoc* analysis revealed that prenatal stress increased licking duration in males only in the second phase compared to non-stressed males (Figure 2C) whereas stressed females showed higher rates of licking than did non-stressed females in the first phase, the interphase and at the peak of the second phase (Figure 2D).

#### Drug Effects in Prenatally Stressed Rats (Figure 3)

For flexing and shaking, the *post hoc* analysis revealed that for males, both drugs and their combination decreased flexing + shaking behavior in the first and the second phases compared with vehicle control males (Figure 3A); in addition, B-PS and FB-PS decreased flexing + shaking behavior in the interphase. In females, neither of the drugs nor their combination significantly changed flexing + shaking behavior (Figure 3B).

For licking duration, the *post hoc* analysis revealed that in males both drugs and their combination decreased licking duration in the second phase; in addition, B-PS decreased licking in the first phase (Figure 3C). In females, both drugs and their combination decreased licking in the second phase compared vehicle stressed females; also, B-PS decreased licking in the first phase and interphase and FB-PS in the first phase only (Figure 3D).

#### Comparison Between Drug Effects, Sex Differences (Figure 4)

For flexing and shaking, the antinociceptive effect of B-PS was greater that of FB-PS in males and females during the interphase (Figures 4A,B), and in females, in the second half of the second phase (Figure 4B). B-PS was more effective than F-PS during the interphase in males and females (Figures 4A,B). Sex differences in flexing + shaking behavior were found in FB-PS rats, in the both phases and the interphase (Figure 4B) with a stronger antinociceptive effect in males.

For licking duration, antinociceptive effect of B-PS was greater that of FB-PS in the interphase in males and females (Figures 4C,D), and greater than that of F-PS in the first phase, the interphase and the second phase in females (Figure 4D). The antinociceptive effect of FB-PS was greater that of F-PS in the first phase in females (Figure 4D). Sex differences in licking duration were found in the interphase in F-PS and FB-PS with the stronger antinociceptive effect in males (Figure 4D).

## Forced Swim Test (Figure 5)

For immobility time, there were significant effects of Treatment and *post hoc* analyses for males and females combined showed that prenatal stress resulted in increased time immobile and that all three drug conditions reduced immobility to control levels. Identical results were found when each sex was analyzed separately. Thus, stress alone increased the time of immobility significantly in males and females, and this was decreased significantly by fluoxetine, buspirone or combination of the drugs in prenatally stressed male and female rats.

**Figure 5:**
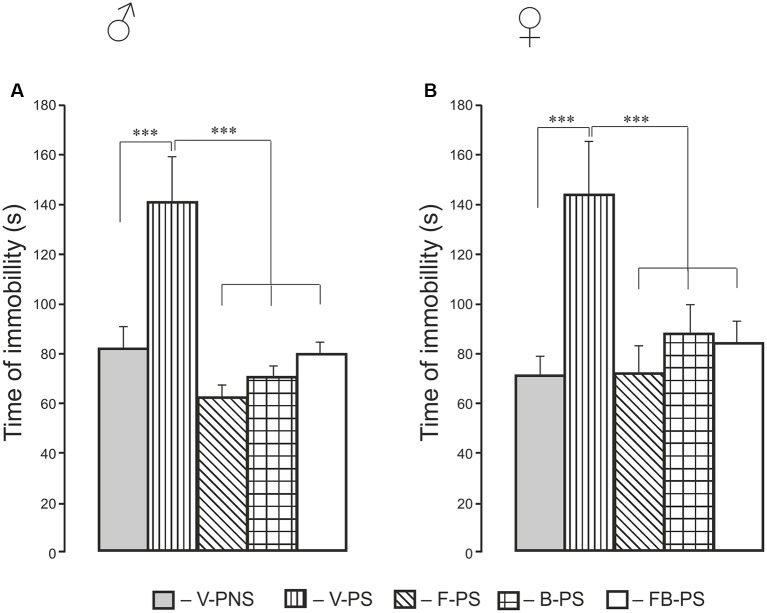
Effects of prenatal stress, fluoxetine, buspirone and their combination on the time of immobility in the forced swim test in young male **(A)** and female **(B)** rats. V-PNS, vehicle without prenatal stress (without stress of pregnant dams), V-PS, vehicle and prenatal stress, F-PS, fluoxetine and prenatal stress, B-PS, buspirone and prenatal stress, FB-PS, combination of fluoxetine and buspirone and prenatal stress. ***p < 0.001. Data are mean ± one SEM.

### Corticosterone Determination (Figure 6)

**Figure 6:**
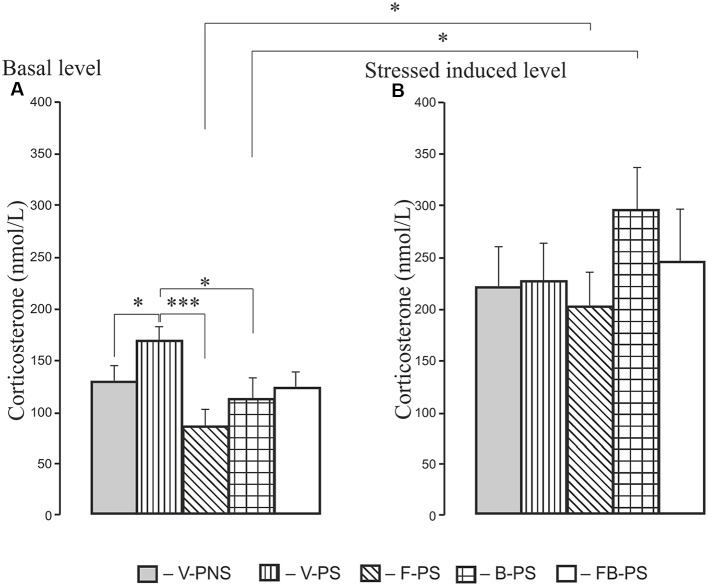
Basal level of corticosterone **(A)** and level of corticosterone after forced swimming **(B)** in 25-day-old male rats. For Basal: *p < 0.05, ***p < 0.001. For Forced Swimming vs. Basal: *p < 0.05. Data are mean ± one SEM.

For basal corticosterone, there was the main effect of Treatment and *post hoc* analyses revealing a stress induced increase in basal level of corticosterone in prenatally stressed rats. F-PS and B-PS decreased the basal level of corticosterone below that of the vehicle stressed animals (Figure 6A). Forced swimming increased corticosterone level in stressed rats given fluoxetine, buspirone compared with the basal level of corticosterone in rats with corresponding prenatal injections. There were no significant differences in the level of the hormone between F-PS, B-PS, FB-PS, V-PS and V-PNS rats (Figure 6B).

## Discussion

### Summary of Findings

The present study presents novel data that describe the effects of repeated prenatal administration of fluoxetine, buspirone or their combination on pain- and depressive-like behavior that were enhanced by prenatal stress. Prenatal stress disrupts the normal development of the serotonergic system (Van den Hove et al., 2006; Gemmel et al., 2018; Kiryanova et al., 2018; Soares-Cunha et al., 2018), the HPA axis (Weinstock, 1997; Gemmel et al., 2017; Morsi et al., 2018), and the brain morphology (van den Bergh et al., 2018). Likewise, the drugs we used act through the 5-HT1AR (Albert and Fiori, 2014) and likely alter its development (Lauder et al., 2000). Our results demonstrate that prenatal stress increased basal thermal pain sensitivity, prolonged formalin-induced pain-like response, and depressive-like behavior in the young rats of both sexes. The time-course of formalin-induced pain showed sexual dimorphism in response to prenatal stress. Prenatal treatment with the antidepressant fluoxetine, the anxiolytic buspirone or their combination eliminated the adverse influences of prenatal stress on pain and depressive-like behavior in male and female rats. For the acute thermal pain assay, for males B-PS and FB-PS reversed the effects of stress with FB-PS being much more effective than either drug alone. For females, the results were similar except that B-PS and FB-PS were equally effective. In the first acute pain phase of the formalin test the stress induced elevated flexing and shaking was reduced equally by all three drug treatments only in males. These treatments had no effect on these behaviors in females. For licking, the acute pain response was decreased in males only by B-PS, whereas in females both B-PS and FB-PS were effective. In the interphase for flexing + shaking and licking behavior, the antinociceptive effect of B-PS dominated that of FB-PS and that of F-PS in the interphase for flexing + shaking behavior in rats of both sexes. In the second, inflammatory phase of the formalin test, stress-induced increases in flexing and shaking and licking were reduced by drug treatment in males and only in licking in females. However, B-PS was more effective than F-PS in reducing licking behavior in all three phases of the formalin test only in females. In the Forced swim test, there was not the expected advantage in effects of the antidepressant combination of FB-PS compared to those of F-PS, in both sexes.

### Prenatal Stress Effects

Prenatal stress increased basal thermal pain sensitivity as evidenced by a decreased the paw withdrawal latency in the hot plate test equally in both male and female rats; these results support our previous data (Mikhailenko and Butkevich, 2019). The time-course of formalin-induced pain in response to prenatal stress, to the contrary, showed marked differences between male and female rats with prenatal stress failing to increase flexing and shaking compared to non-stressed females. In addition, the effect of prenatal stress on flexing + shaking behavior during the first acute phase was more intense in males than in females. The increase of the first phase in V-PS males, but not in V-PS females, may be associated with a differential influence of prenatal stress on nociceptors *via* microglia. According to the current literature, prenatal stress activates microglia, which releases cytokines and chemokines (Cattane et al., 2018), that can directly sensitize nociceptors and thereby directly mediate pain during inflammatory conditions (Cook et al., 2018). There are sex differences in the prenatal development of microglia, which in turn may differentially program of sexual differences in the brain and behavior (Nelson and Lenz, 2017; VanRyzin et al., 2018). These putative mechanisms will require further study.

We also found that in the second tonic phase of the formalin test, prenatal stress increased licking, organized at the supraspinal level, in the rats of both sexes, but flexing + shaking behavior, organized at the spinal level, differed significantly only in males. The second phase in the formalin test is believed to be due to central sensitization of spinal neurons, induced by stimulation of TRPA1 during the first phase, and by an inflammatory response triggered by an “inflammatory soup” consisting of many mediators (serotonin, prostaglandins, IL-1β, IL-6 etc.; Basbaum and Jessell, 2000). The interphase and the second phase of formalin-induced pain are also modulated by descending inhibition from the periaqueductal gray (PAG), projecting to the rostral ventromedial medulla (RVM) and subsequently to the spinal cord (Millan, 2002). This constitutes a major mechanism by which pain transmission is modulated (Fields and Basbaum, 1999). These descending pathways from the RVM are the main source of serotonergic inputs to the spinal dorsal horn, and the alteration by prenatal stress of serotonergic system function in a sex-specific manner may explain the different effects of prenatal stress in male and female rats. This hypothesized mechanism is supported by previous findings demonstrating sexual dimorphism in anatomical organization and the functional activation of the PAG-RVM circuit (Loyd and Murphy, 2006).

### Effects of Prenatal Drug Treatment

#### Thermal Test

We found that prenatally stressed male and female rats treated with buspirone or combination of the drugs mitigated the effects of prenatal stress in the hot plate test. The antinociceptive effect of FB-PS was significantly greater than that of F-PS alone in both males and females, but there was no significant antinociceptive effect of fluoxetine. There are few existent data on the effects of repeated fluoxetine treatment on pain-like behavior; in non-prenatally stressed adult guinea pigs, there were increased thermal pain thresholds (antinociceptive effect; Vartazarmian et al., 2005); in prenatally stressed adult male rats, post-operative pain measured as hypersensitivity to mechanical stimuli after hind paw incision at 56 days of age, was normalized by perinatal fluoxetine (Knaepen et al., 2013). In the clinic, perinatal maternal stress increases pain sensitivity in infant children that is ameliorated by prenatally exposure to SSRI’s (Oberlander et al., 2005).

#### Formalin Test

Although both drugs and their combination attenuated the effects of prenatal stress on formalin-induced pain, there were differences in these effects on the prolonged biphasic response. Importantly, the drugs were effective only when formalin-induced pain was altered by prenatal stress. Both the preclinical and clinical data on the antinociceptive effects of fluoxetine in adults are inconsistent. A recent review of the literature concluded that the most likely beneficial use of fluoxetine in nociceptive pain management is for the alleviation of inflammatory pain (Barakat et al., 2018). Fluoxetine does not possess significant analgesia effects on its own (Hamdy et al., 2018). Both studies indicate that if fluoxetine is combined with morphine it does not enhance acute morphine analgesia but rather attenuates opioid tolerance and dependance. This is consistent with the hypothesis that although fluoxetine itself may be a weak or ineffective analgesic, it may modulate enhanced pain induced by other means perhaps in close conjunction with the descending serotonergic regulation (Basbaum, 1981).

In the test of formalin-induced pain, fluoxetine reduced the total time spent licking and flinching to control levels in the second phase in females but not males (Zammataro et al., 2017). In contrast, F-PS, B-PS and FB-PS decreased flexing + shaking behavior in the second phase in prenatally stressed males but not females. Again, the imposition of stress on the rats altered the effects of fluoxetine on pain in a sex-dependent manner.

Of particular interest, it was the interphase that proved most sensitive to prenatal influence of B-PS as compared with that of FB-PS and F-PS in flexing + shaking and licking behaviors in both sexes (Figure 4). These data suggest that buspirone, to which pregnant stressed dams were exposed, had the greatest effects on the developing descending serotonergic inhibitory system, modulating nociceptive signals in spinal cord dorsal horn neurons. Buspirone is used in the clinic as a serotonergic anxiolytic in patients with increased anxiety and may have antidepressant properties (Savitz et al., 2009; Albert and François, 2010; Kirilly et al., 2015; Haleem et al., 2018), which would be consistent with our data here. Long-term treatment with buspirone downregulates 5-HT1A autoreceptors and enhances serotonergic functions *via* postsynaptic 5-HT1A heteroreceptors in the hippocampus, cortex and limbic system (Savitz et al., 2009) to mediate the antidepressant actions of 5-HT and reduce pain perception. Negative feedback is speculated to play a major role in the delayed action of SSRI antidepressants because it takes time to overcome the autoreceptor down-regulation.

### Potential Mechanisms

The properties of buspirone as a full agonist of presynaptic and partial agonist of postsynaptic 5-HT1A receptors determine its use in combination with other antidepressants to increase efficacy and reduce side effects in the treatment of depression (Albert and François, 2010; Albert and Fiori, 2014; Stuivenga et al., 2019). When using the combination of fluoxetine and buspirone, the increased extracellular concentration of 5-HT (the action of fluoxetine) occurs without decreased sensitivity of the postsynaptic 5-HT1A receptors due to the ability of the partial agonist buspirone to reduce the function of 5-HT1A autoreceptors. This results in a greater release of 5-HT by fluoxetine by reducing the negative feedback mechanisms mediated by 5-HT1A autoreceptors and increasing the stimulation of the postsynaptic 5-HT1A receptors (Pierz and Thase, 2014; Wang et al., 2015; Stuivenga et al., 2019).

A comparative analysis of basal plasma corticosterone level in males confirmed the effectiveness of prenatal repeated administration of drugs: prenatal stress increased the basal corticosterone level compared to controls and prenatal drug administration reversed that increase. There were no significant differences in the stressed induced level of the hormone between F-PS, B-PS, FB-PS rats and V-PS and V-PNS rats, probably due to the high reactivity of the HPA axis in response to the severe physical and psycho-emotional stress of forced swimming. Fluoxetine can be immunomodulatory, acting on serotonergic neurons in the CNS and regulating neuroendocrinal signals (Di Rosso et al., 2016). Fluoxetine normalizes the immune function, increasing it when conditions reduced function, but decreasing it under conditions of high function (for example, in prenatally stressed rats). Because of the anti-inflammatory properties of fluoxetine (Valera et al., 2014) and buspirone (Sharifi et al., 2015), we cannot exclude the hypothesis that their behavioral effects as shown here are due to normalization inflammatory cytokines. Cytokines contribute to the restoration of afferent-efferent connections of the raphe nuclei with the PFC and spinal cord, which are involved in the integration of antinociceptive and psycho-emotional systems (Wang and Nakai, 1994). If stress during pregnancy has deleterious effects on the fetal immune system (Lasselin et al., 2019), the improvement of the adaptive behaviors studied here in the prenatally stressed rats by prenatal exposure to buspirone, fluoxetine or their combination can be viewed as a manifestation of their protective properties on immune function. Whether these findings will hold true for females remain to be tested.

### Sex Differences

The benefits of FB-PS over F-PS alone were seen in females only in the first acute phase of licking behavior when FB-PS normalized the stress-induced pain response, whereas F-PS had no effect. Considering that fluoxetine and buspirone act *via* 5HT1AR, these results point to differences in the mechanisms by which they act prenatally on the development of 5-HT1AR. Of particular interest, the antinociceptive effects of FB-PS were greater in males than females in the first phase, the interphase and the second phase for flexing + shaking behaviors and in the interphase in FB-PS and F-PS for licking behavior. Thus, the combined drug treatment of prenatally stressed pregnant dams more effectively attenuated inflammatory pain-like behavior in male offspring than the female offspring. It will be particularly important to extend these findings to post-sexual maturity subjects to understand the interplay between prenatal stress, sex and transitions across pre-adolescence, adolescence and post-adolescence. It is during this time that the prevalence and risk of psychiatric disease is rapidly changing and when sexually dimorphic chronic pain conditions first emerge (Paus et al., 2008).

Sexual dimorphism found in the prenatally stressed rats in pain-related indices is associated with suppression of the release of testosterone, which itself has an analgesic effect (Edinger and Frye, 2005; Da Silva et al., 2018). The sex steroid hormones estrogens and androgens modulate prenatal and postnatal development of many processes including the nociception, the HPA axis and immune system (Green and McCormick, 2016; Fanton et al., 2017). Differences in the prenatal action of the drugs on inflammatory painful behavior may also be related to the sexual differences in the development of microglia during critical periods prenatally (Nelson and Lenz, 2017). These sex-dependent differences in the course of microglial development may determine the different sensitivity of microglia to the effects of fluoxetine and the combination of the drugs in the two sexes (Schwarz et al., 2012), as the immune system acts as a regulator of sex differences in brain development and behavior (Nelson and Lenz, 2017; VanRyzin et al., 2018; Goldstein et al., 2019). Current genetic studies emphasize that the epigenetic and behavioral effects of prenatal environmental exposures are often found to be sex-specific (Kundakovic and Jaric, 2017). Differences in drug susceptibility, reactions to stress, may be due to polymorphism of the 5-HT1A gene (Albert and Fiori, 2014; Luckhart et al., 2016). These sex differences in 5-HT1A autoreceptor function suggest that different adaptive mechanisms are involved in males and females to regulate 5-HT activity and behavior. Among the transcription factors that suppress the expression of the 5-HT1AR gene in the raphe nuclei is the Deaf-1 factor transcription regulator, which increases serotonergic tone by suppressing the presynaptic expression of 5-HT1AR and simultaneously stimulating postsynaptic 5-HT1AR (Albert and François, 2010; Albert and Fiori, 2014).

In summary, our results show that the inflammatory pain-like responses organized at the spinal level in young pre-adolescent males are more vulnerable to prenatal stress and more sensitive to prenatal drugs compared to pre-adolescent young females. These results re-emphasize the importance of studying both sexes to study the mechanisms of long-term prenatal influence of drugs. Considering the lack of clinical and experimental data on the effects of antidepressants used to treat depression during pregnancy on the nociceptive system’s vulnerability to inflammatory agents in the offspring, the results of this work will be useful for studying the mechanisms of action of the studied drugs and also for predicting analgesics dosing in male and female offspring of mothers exposed to stress and antidepressants during pregnancy.

## Ethics Statement

All experimental procedures were approved by the Local Ethics Committee for Animal Experiments of the I. P. Pavlov Institute of Physiology, Russian Academy of Sciences (St. Petersburg, Russia) and followed the guidelines published by the Committee for Research and Ethical Issues of the IASP on ethical standards for investigations of experimental pain in animals.

## Author Contributions

IB and VM: experimental design. IB, VM and EV: collection of data, conduction of statistical analyses. IB, VM, EV and GB: interpretation and analysis of data, participated in the drafting and revising of the manuscript and reviewed and approved the final submitted manuscript.

## Conflict of Interest Statement

The authors declare that the research was conducted in the absence of any commercial or financial relationships that could be construed as a potential conflict of interest.
